# Epigenetic dysregulation in cardiovascular aging and disease

**DOI:** 10.20517/jca.2021.16

**Published:** 2021-08-23

**Authors:** Allison B. Herman, James R. Occean, Payel Sen

**Affiliations:** Laboratory of Genetics and Genomics, National Institute on Aging, NIH, Baltimore, MD 21224, USA.

**Keywords:** Epigenetics, chromatin, cardiovascular, senescence, aging

## Abstract

Cardiovascular disease (CVD) is the leading cause of mortality and morbidity for all sexes, racial and ethnic groups. Age, and its associated physiological and pathological consequences, exacerbate CVD incidence and progression, while modulation of biological age with interventions track with cardiovascular health. Despite the strong link between aging and CVD, surprisingly few studies have directly investigated heart failure and vascular dysfunction in aged models and subjects. Nevertheless, strong correlations have been found between heart disease, atherosclerosis, hypertension, fibrosis, and regeneration efficiency with senescent cell burden and its proinflammatory sequelae. In agreement, senotherapeutics have had success in reducing the detrimental effects in experimental models of cardiovascular aging and disease. Aside from senotherapeutics, cellular reprogramming strategies targeting epigenetic enzymes remain an unexplored yet viable option for reversing or delaying CVD. Epigenetic alterations comprising local and global changes in DNA and histone modifications, transcription factor binding, disorganization of the nuclear lamina, and misfolding of the genome are hallmarks of aging. Limited studies in the aging cardiovascular system of murine models or human patient samples have identified strong correlations between the epigenome, age, and senescence. Here, we compile the findings in published studies linking epigenetic changes to CVD and identify clear themes of epigenetic deregulation during aging. Pending direct investigation of these general mechanisms in aged tissues, this review predicts that future work will establish epigenetic rejuvenation as a potent method to delay CVD.

## INTRODUCTION

Cardiovascular disease (CVD), including heart failure, hypertension, atherosclerosis, and cardiomyopathy, remains the leading cause of death worldwide and carries a severe socioeconomic burden. While many factors contribute to CVD development, including diet, genetics, and the environment, one core, independent risk factor amongst almost everyone with a CVD is aging. It was once estimated that by 2030, about 20% of the United States population would be aged 65 or older and that CVD will account for 40% of the deaths of that group, making CVD the leading cause of death^[1,2]^. According to the United States Census Bureau, we have observed an increase in the elderly population from 40 million in 2007 to 51 million in 2017, with the projected number of people over 65 to leap to 95 million in 2060. CVD prevalence continues to increase as human life expectancy also continues to rise, likely due to greater exposure to the traditional external risk factors and intrinsic pathways of aging^[3]^. After adjusting for the other major risk factors for CVD, one study found the odds of vascular diseases increased with every decade of life, demonstrating a strong increase in peripheral arterial disease (PAD), carotid artery stenosis, and abdominal aortic aneurysm (AAA) with advanced age^[4]^.

The pathological consequences associated with normal cardiovascular aging include hypertrophy, altered left ventricular (LV) diastolic and systolic function, heart failure, enhanced arterial stiffness, and endothelial dysfunction, all of which can alter the structure and function of the heart and arterial system^[5,6]^. In the vasculature specifically, aging contributes to decreasing vascular compliance. Furthermore, it promotes vascular remodeling, including calcification and fibrosis, which in turn precedes the development of hypertension and accelerates the progression of other vascular-related diseases such as atherosclerosis or heart failure^[7]^. In addition, the incidence of metabolic diseases such as diabetes also increases significantly with age and contributes greatly to CVD morbidities and mortalities^[8]^. Interestingly, many metabolic disorders are associated with premature aging, suggesting that there are mechanisms we can unravel to potentially intervene and prevent the deterioration of the cardiovascular system independent of natural aging.

Until recently, aging has widely been considered an unmodifiable risk factor for many chronic diseases (cancer and neurodegenerative diseases) and very prominently CVDs^[9–11]^. Aging interventions have become a rising area of interest, where molecular and clinical dissection of aging processes have begun to show promising therapeutic targets. The monumental finding in 1939 that caloric restriction (CR) in mice and rats, and most recently in primates, extended lifespan led to the important hypothesis that lifespan extension with delayed aging improved healthspan^[12,13]^. Since then, an examination of healthy aging and processes that promote age-related deterioration across species and organs has increased our understanding of the involvement of aging in chronic diseases^[14]^.

## THE ROLE OF SENESCENT CELLS IN CARDIOVASCULAR PATHOLOGIES AND AGE-RELATED PATHWAYS

### CVD and cellular senescence

A major contributor to age-related cellular dysfunction was found to be the accumulation of senescent cells in tissues^[15]^. Senescent cells were discovered in 1965 by Hayflick^[16]^ as cells with a limited proliferative capacity; however, we now define senescence as those cells with indefinite cell cycle arrest, resistance to apoptosis, and expression of a senescence-associated secretory phenotype (SASP)^[17]^. Thus, senescent cells provide a new avenue for therapeutic interventions, known as senotherapies^[18,19]^. Specifically, a class of drugs known as “senolytics” is designed to take advantage of the senescent cell’s resistance to apoptosis by targeting cell survival pathways to eliminate senescent cells from tissue selectively, thereby removing their detrimental effects^[20–22]^. Alternatively, another class of drugs known as “senostatics” is designed to modulate the proinflammatory SASP; however, the complex composition of the SASP varies widely among different cell types, different stages of senescence (early, middle, or late), and various senescence inducers, providing many obstacles to a successful therapeutic intervention^[23]^. While the components of the SASP may vary, the beneficial effects of senotherapies (both senolytics and senostatics) are mostly attributed to blunting the secretion of proinflammatory cytokines, chemokines, growth factors, and extracellular matrix (ECM) remodeling proteins, among others secreted by senescent cells^[24–26]^. For a more detailed discussion of these senotherapeutic agents, please consult the reviews by Kirkland and Tchkonia^[24]^, 2020 and Robbins *et al*.^[26]^, 2021.

There is a rapidly growing body of evidence supporting the deleterious role of senescent cells in several CVDs. During embryonic development, tissue regeneration, and wound healing, vascular senescent cells have a beneficial presence to maintain homeostasis^[27]^; however, we have learned that impaired removal and accumulation of senescent cells in cardiovascular tissue foments impaired function and disease development. Senescent cells have been implicated in several CVD pathologies, most notably, atherosclerosis^[28]^, AAA^[29]^, cardiac fibrosis^[30]^, heart failure^[31]^, and hypertension^[32]^. Further incriminating senescent cells as causative agents of CVD, Childs *et al*.^[33]^ demonstrated that senescent cells are critical drivers of atherosclerosis and selective removal of these cells has therapeutic potential to improve disease outcomes. In the same year, Roos *et al.*^[34]^, found that pharmacological clearance of senescent cells can lessen the vasomotor dysfunction that occurs in murine aging and atherosclerosis.

### Senescent cardiomyocytes contribute to cardiac pathologies

Heart failure is an age-related cardiac pathology that is a major source of mortality, affecting approximately 1% of all people over 50 years and doubling its prevalence with each decade of life^[35,36]^. Cardiomyocyte senescence is common in cardiac aging and related diseases, although senescent cardiomyocytes are more difficult to identify due to their terminally differentiated state^[37]^. Senescent cardiomyocytes display contractile dysfunction, endoplasmic reticulum (ER) stress, DNA damage, genomic instability, declining mitochondrial function, SASP, and hypertrophic growth^[38]^. Further, the exact triggers and effects of cardiomyocyte senescence *in vivo* have not been well described. However, studies in mice and rats have identified many of the signatures of cellular senescence, such as increased cardiomyocyte size, telomere attrition, ROS production, and elevated senescence markers p16 (CDKN2A) and p53 (TP53)^[39,40]^. In hypertrophic cardiomyopathy patients, cardiomyocytes with DNA damage also had the shortest telomeres, and patients with ischemic cardiomyopathy also displayed shortened telomere length^[41]^. While senescence is often associated with telomere shortening, cardiomyocytes are post-mitotic cells that do not experience replicative exhaustion; therefore, senescent cardiomyocytes demonstrate length-independent telomere damage caused by mitochondrial dysfunction and ROS^[42]^. As mentioned above, hypertrophy is a hallmark of age-associated heart dysfunction, and although cardiomyocyte hypertrophic growth is commonly associated with senescence, it is unclear whether senescent myocyte growth directly contributes to cardiac hypertrophy^[43]^. A few studies have found that ER stress appears to promote a hypertrophic cardiomyocyte phenotype *in vitro*, hypertrophy was detected in hearts post-infarction, and aged rat hearts demonstrated cardiomyocyte hypertrophy and increased LV fibrosis; however, none of these studies directly measured senescence^[43–45]^. Interestingly, treatment of aged mice with the senolytic drug navitoclax selectively removed senescent cardiomyocytes, which improved myocardial remodeling and increased survival following myocardial infarction^[46]^. While studies have outlined that accumulated damage to mitochondria, proteins, and DNA with age contributes to cardiomyocyte malfunction, telomere damage and cellular senescence are also critical to heart failure in humans, and more efforts will be needed to fully elucidate the contribution of senescent cardiomyocytes to age-related cardiac pathologies^[47]^.

Further, cardiomyocyte senescence and the downstream pathologies are also the results of stress-induced premature senescence. Cardiomyocytes treated with doxorubicin demonstrated similar characteristics to those of aged rats, including increased senescence-associated beta-galactosidase positive cells, reduced telomerase activity, and increased expression of cell cycle regulatory proteins such as p16 and p21 (CDKN1A)^[48]^. Recently, Mitry *et al*.^[49]^, further described the mechanism by which doxorubicin accelerates cardiomyocyte senescence and cardiotoxicity. In the study, Mitry *et al*.^[49]^ found that doxorubicin caused early and persistent topoisomerase-induced mtDNA damage that enhanced cardiomyocyte senescence, in turn straining the heart’s aerobic metabolism over time and promoting late-onset heart failure often observed in survivors of childhood cancers.

Aside from cardiomyocytes, other cardiac cells promote senescence and aging and the downstream age-related diseases. For example, cardiac fibroblasts secrete many paracrine factors such as matrix metalloproteinases and express integrins to promote signaling and ECM interactions that regulate cardiomyocyte senescence^[50,51]^. Endothelial cell senescence has also been implicated in heart failure with preserved ejection fraction, in which the activation of p53 signaling generates cardiac inflammation and left ventricular pressure overload in mice^[52]^. Interestingly, cardiomyocyte dysfunction can also promote changes in neighboring cell types, such as fibroblasts, and impair the reparative function of cardiac fibrosis after cardiomyocyte injury^[53,54]^.

An important area of study for cardiac aging is impaired cardiomyocyte regeneration. We have discussed the key hallmark of cardiac aging; the increased size of cardiomyocytes, but another critical change is the loss of cardiomyocytes with age^[55]^. While the neonatal heart demonstrates regenerative capacity, it was long thought the adult heart lacked the ability to renew cardiomyocytes^[56]^. Recent observations that adult cardiomyocytes renew at a rate of 0.5% to 2% per year demonstrating a limited, innate regenerative ability that has dismantled those previous theories; however, the capacity of the heart to regenerate declines with age^[57–59]^. Increased cardiomyocyte death, even in the very small numbers, was shown experimentally to promote heart failure, and that inhibiting the loss of cardiomyocytes, potentially through regeneration, could be an ideal therapeutic avenue^[60]^. The heart regeneration field suffers from a lack of consistent and reproducible data on the subject; however, a consensus has developed that stem cells are not the source of cardiomyogenesis, but rather preexisting cardiomyocytes divide to give rise to new cells^[57,61–63]^. A deeper understanding of the mechanisms that drive cardiomyocyte death with age may yield therapeutic potential for promoting regeneration in aged and damaged hearts.

### Senescent vascular cells contribute to vascular diseases of aging

Among the many changes observed with aging, arterial remodeling and dysfunction are critical to the development of CVD, even in individuals who may be deemed healthy by all other standards. For example, aged arteries are defined by an increased ratio of intima-to-media thickness, and multiple reports have determined a 2- to 3-fold increase in intima thickness between 20- and 90-year-old people^[5]^. In addition, changes in the arterial wall feature increased collagen synthesis and elastin degradation with age, promoting arterial stiffness and reduced elasticity^[64]^. The consequence of such vascular remodeling manifests as increased blood pressure and lower diastolic pressure generating a predisposition to developing hypertension and atherosclerosis, among other vascular diseases^[65–67]^.

Both of the primary cell types of the artery, vascular smooth muscle cells (VSMCs) and endothelial cells (ECs), become senescent with age, regardless of the presence of a vascular-related disorder^[68–70]^. The human VSMCs from aged vessels and advanced-stage atherosclerotic plaques displayed senescence indicators with prolonged population doubling times and reduced cell proliferation^[71,72]^. These findings were corroborated by associating the growth arrest of VSMCs with increased expression of p16 and p21, cyclin-dependent kinase inhibitors, and RB1 phosphorylation, all of which are observed during replicative VSMC senescence and are widely considered hallmarks of senescence^[28,73,74]^. The VSMCs from the fibrous cap region of the atherosclerotic plaque compared to the vascular media demonstrated telomere shortening caused by oxidative stress-induced DNA damage. The resulting VSMC senescence accelerates vascular disorders such as atherosclerosis^[28]^. Angiotensin II is another well-described driver of VSMC senescence, and recently, smooth muscle 22α, an actin-binding protein, has been shown to prevent p53 degradation via MDM2 suppression to promote angiotensin II-induced VSMC senescence^[75]^. Importantly, senescent VSMCs in the plaque of carotid arteries express enhanced levels of interleukin-6 (IL-6), signifying VSMCs as a SASP producer and source of inflammation during vascular disease^[76]^. Most recently, Uryga *et al*.^[77]^ suggested persistent telomere damage in VSMCs causes senescence and inflammation via immune cell recruitment and retention. Overall, senescent VSMCs have been recognized in atherosclerotic lesions, AAA, and PAD, suggesting that VSMCs have a critical role in age-related vascular pathologies^[70]^.

Aside from VSMCs, ECs play an influential role in vascular disorders with age. While ECs typically maintain vascular homeostasis, senescent or dysfunctional ECs establish proinflammatory, prothrombotic, and vasoconstrictor characteristics in addition to reduced proliferation and migration. Replicative senescent ECs express increased cell adhesion molecules such as ICAM-1 and decreased endothelial nitric oxide synthase and activity, caused by telomere shortening^[70]^. Another cause of EC senescence may be disturbed flow during atherosclerosis. In both mice and *in vitro*, the aberrant flow was a driver of EC senescence by activating the p21-p53 pathway^[78]^. Aged and senescent ECs are also producers of inflammatory cytokines, namely IL-6, tumor necrosis factor alpha (TNFα), and monocyte chemoattractant protein-1 (MCP-1), which also suggests that the accumulation of senescent ECs in the artery with age causes chronic sterile inflammation and vascular changes that predispose one to vascular diseases^[79,80]^.

**Figure 1.**
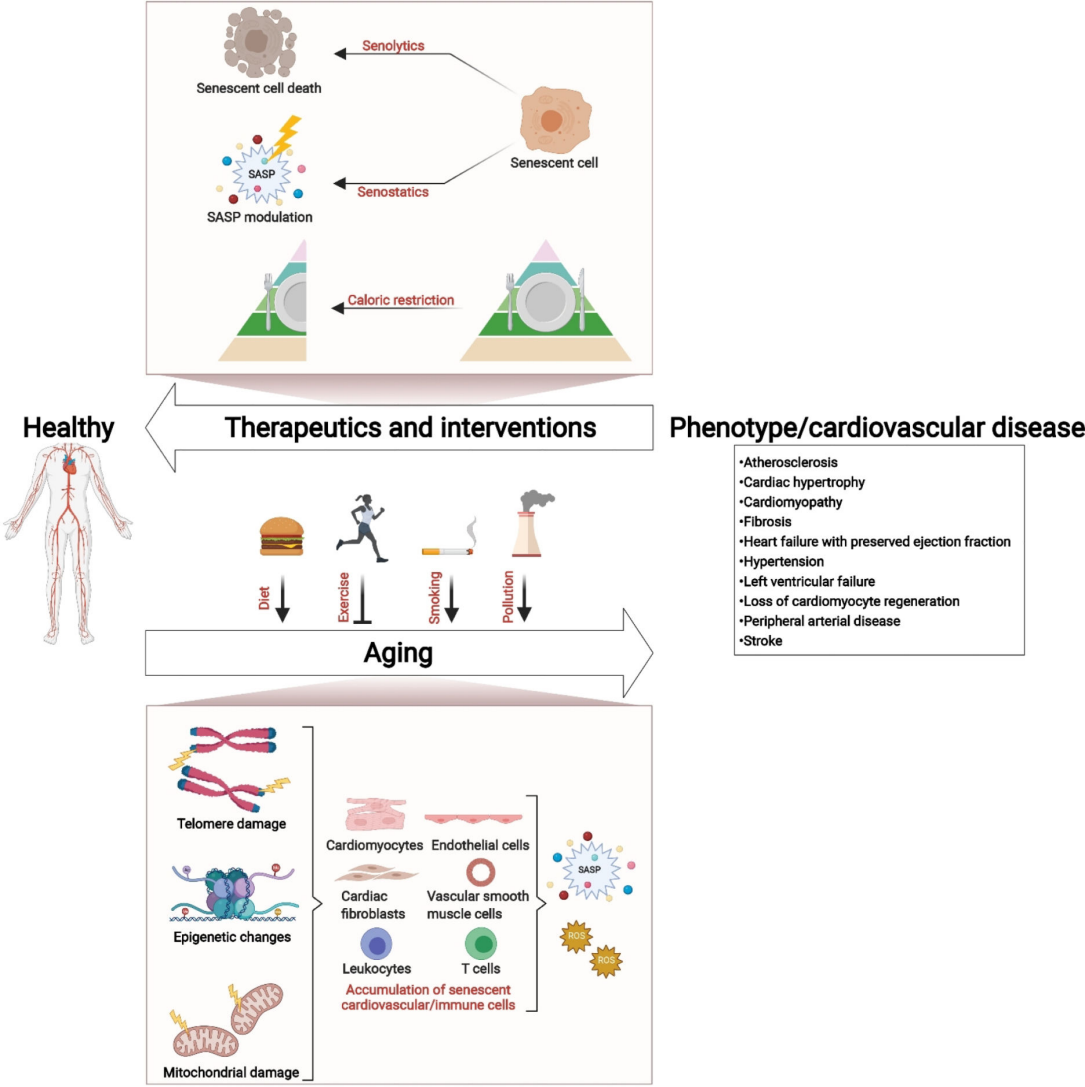
Molecular mechanisms involved in cardiovascular aging: consequences and potential therapeutics and interventions. Telomere damage, epigenetic changes, and mitochondrial damage are associated with the accumulation of senescent cardiovascular/immune cells, cardiovascular aging, and disease. Diet, smoking, and air pollution can also negatively contribute to aging, while physical exercise may improve cardiovascular health. Potential therapeutics and interventions include targeted elimination of senescent cells (senolytics), modulation of the proinflammatory SASP (senescence-associated secretory phenotype; senostatics), and dietary interventions (caloric restriction).

Although VSMCs and ECs compose most of the artery, immune cell aging and senescence may also contribute greatly to vascular pathologies associated with aging. Individuals 60 years or older with shortened telomeres in leukocytes experience a higher mortality rate that has been linked to increased death from CVD^[81]^. In an interesting and clinically relevant study, the analysis of leukocyte populations led to the finding that telomere length was strongly associated with the development of atherosclerosis and CVD^[82,83]^. Furthermore, senescent leukocytes and senescent effector memory T cells were found preferentially in unstable atherosclerotic plaques^[84]^. Additionally, enhanced cytokine expression (TNF, MCP-1/CCL2, IL6) and ROS production have been observed in monocytes from atherosclerosis patients^[85]^. Importantly, the proinflammatory phenotype of aged and senescent monocytes is driven by senescence^[86]^. Figure 1 summarizes the known consequences of cardiovascular aging and the molecular mechanisms, cell types, environmental factors involved, and potential therapeutics and interventions.

Overall, the evidence overwhelmingly points to the need to continue to study aging and senescence in CVD. Here, we present the body of work thus far that has uncovered numerous important pathways and mechanisms by which aged and senescent cells contribute to the development of different cardiovascular pathologies. Recurring thematic features of cardiovascular aging and disease suggest an unstable genome with shortened telomeres and a deregulated transcriptome that is pro-fibrotic, proinflammatory, but anti-proliferative with reduced regenerative capacity. In sum, these cellular phenotypes suggest an altered epigenome that has emerged as one of the hallmarks of aging in recent years. By focusing on mechanisms with druggable targets such as epigenetic alterations, we can develop therapies to modulate aging and senescence in CVD. Outlined below are the central findings from studies that have investigated epigenetic changes in CVD, although as discussed in FUTURE PERSPECTIVES, these studies are limited and mostly out of context with aging. Nevertheless, these studies have revealed important insights that can be validated and developed into targeted therapeutics in the future.

## EPIGENETIC CHANGES IN THE AGING CARDIOVASCULAR SYSTEM

Epigenetic alterations are one of the key features of aging and age-related disease, including CVD. These alterations include changes in DNA modifications, histone modifications, histone composition, transcription factor (TF) binding, non-coding RNA-mediated regulation, chromatin remodeling, nucleosome positioning, and 3D genome folding^[87,88]^. Whether epigenetic changes drive aging or are a consequence of activated stress signaling pathways remains to be dissected but likely are part of a vicious cycle that ultimately leads to tissue damage, inflammation, and disease. In the following sections, we first discuss the probable impact of diet, exercise, and other environmental factors on cardiovascular health and then elaborate on the role of specific epigenetic regulators studied in the context of cardiovascular aging and disease.

### Effect of diet, exercise, and other environmental factors in cardiovascular health

Aside from age, obesity and metabolic syndrome (characterized by sarcopenic obesity, insulin resistance, inflammation, *etc*.) are also major risk factors for CVD, partly due to their systemic proinflammatory effects, much like in aging^[89]^. With increased body mass, there is an increase in the overall size of the heart, concentric hypertrophy, increased left ventricular mass, hypertension, and diastolic dysfunction, partially overlapping age-related cardiac symptoms. CR (i.e., a reduction in daily calorie intake without malnutrition) is one of the most reproducible lifestyle interventions that improve cardiovascular health and increase lifespan in multiple models^[90]^. Studies in non-human primates show that rhesus monkeys on long-term, moderate CR show improvements in metabolic syndrome, including decreased body weight primarily due to loss of fat, decreased visceral fat mass, improved insulin sensitivity, and an altered lipid profile with more cardioprotective high-density lipoprotein compared to ad libitum fed controls^[13,91]^. Interestingly, an inadvertent CR in humans participating in the Biosphere 2 experiment showed tremendous cardiovascular health benefits^[92]^.

Much like CR, exercise has demonstrated effects on cardiovascular health. Physical inactivity is a major contributing factor to age-related disabilities, declining heart health, stroke, cognitive impairment, and frailty primarily due to progressive arterial stiffness^[93]^. Older individuals undergoing regular exercise show increased maximal oxygen consumption rate (VO_2max_)^[94]^. Endurance exercise improves not only VO_2max_ but also early diastolic left ventricular filling and relaxation, peak ejection fraction, and cardiac output. There are also general improvements in vascular physiology and endothelial function^[95,96]^.

Unlike CR and exercise that have health benefits, smoking is a serious risk factor for cardiovascular disease. Smoking is often quantified in “pack-years”, i.e., the number of packs of cigarettes smoked per day multiplied by the number of years an individual has smoked (cancer.gov). In a study of > 13000 participants, Ding *et al*.^[97]^ determined a strong correlation between pack-years, duration, intensity, and cessation time of smoking to deleterious cardiovascular outcomes, with the strongest risk being PAD. In addition, smoking has been shown to directly target the epigenome, altering DNA methylation profiles, specifically 187 CpG sites independently validated in a separate cohort^[98]^. In fact, some mortality predictive DNA methylation clocks, such as GrimAge (discussed below), directly incorporate smoking-related changes through an estimate of pack-years of smoking^[99]^.

Similar to smoking, air pollution is a major contributing factor that accelerates the decline of cardiopulmonary health. A growing body of epidemiological and clinical evidence indicates that ambient particulate matter may directly impact the cardiovascular system, although exact biological mechanisms are unknown^[100]^. In part, the deleterious effects of particulate pollutants may be mediated by oxidative stress and systemic inflammation^[101]^. Therefore, long-term studies focused on elucidating the molecular pathways involved will be critical for designing mitigative approaches.

Given that environmental factors can impact multiple aspects of cardiovascular health and modulate lifespan, we discuss below some of the key molecular mechanisms that might be involved in this process. The epigenome is the interface between the environment and phenotype and, consequently, plays an important role in regulating health and disease.

### DNA modifications in cardiac pathology

Methylation of cytosine (5-methylcytosine or 5mC) is the best-studied and most abundant modification on DNA. The 5mC status of groups of CpGs is associated with disease onset and mortality and therefore serves as the basis for several pan-tissue clocks that have been designed to act as “biological age” estimators. For example, GrimAge^[99]^ and PhenoAge^[102]^, two epigenetic clocks trained on chronological age and blood-based biomarkers, are associated with time to the incidence of CVD events^[103]^. Although a clear mechanistic basis for these clocks is still obscure, the primary genomic regions affected seem to be polycomb targets and those near developmental genes^[104]^. In concordance, a DNA methylome profiling in purified cardiomyocytes of mice undergoing heart failure showed methylation patterns that resembled those in neonates^[105]^. Another independent epigenome-wide association study examining relationships between DNA methylation and incident CVD discovered two CpG modules in human cohorts: one associated with developmental genes and the other with immune functions^[106]^. In keeping with the developmental gene activation theme in diseased hearts, the landscape of 5-hydroxymethylcytosine (5hmC, an oxidative product of 5mC) in cardiomyocytes derived from developing and hypertrophic hearts resemble, in part, a neonate-like signature. It was shown that 5hmC, which is positively correlated with gene transcription, was reduced over mitochondrial genes and increased over enhancers and gene bodies of fetal genes such as *Myh7*, thereby reactivating them^[107]^.

Investigation of DNA methylation in healthy and atherosclerotic lesions from donor-matched aorta samples interestingly revealed focal hypermethylation in the diseased tissue over repeat and non-repeat regions of the genome and in both a CpG and non-CpG context^[108]^. Furthermore, the differentially methylated regions were associated with endothelial and smooth muscle function. A related study in swine, investigating differential methylation in ECs from an athero-susceptible location (inner curvature of the aortic arch) and an athero-protected region (descending thoracic aorta), also identified many hypermethylated sites that were linked to genes related to transcriptional regulation, pattern-specification HOX loci, oxidative stress, and ER stress adaptive pathway^[109]^. Furthermore, 5’UTR hypermethylation exhibited an inverse relationship with gene expression at the HOX loci primarily. These observations contrast with DNA methylation changes in aging^[110]^ or cancer^[111]^, where global hypomethylation over megabase-sized blocks of the genome is the primary feature despite aging being a risk factor for atherosclerosis.

The derepression of repeat elements with retrotransposon activation is a known molecular event in senescence and aging. Evidence in senescent cells and mouse tissues indicates that these non-coding transcripts, generated from repeat elements, in turn, are reverse transcribed and activate an interferon response contributing to a systemic proinflammatory status in aging^[112]^. The overall coverage of 5hmC over repeat elements was shown to decrease during cardiac development but increase in the hypertrophied heart, particularly at long interspersed nuclear elements. This was accompanied by reduced CG methylation and other repressive histone modifications (discussed below), suggesting a consequential activation of these regions in disease^[107]^.

Most genome-wide methylation studies have been done using bead-based arrays, whole-genome bisulfite sequencing, or reduced representation bisulfite sequencing post-bisulfite treatment of DNA. However, these methods fail to distinguish between 5mC or 5hmC and thereby may complicate mechanistic inferences on gene regulation. The recent development of the oxidative bisulfite sequencing (oxBS-seq) method allows for the simultaneous measurement of 5mC and 5hmC at single-nucleotide resolution^[113]^. We propose that investigation of these distinct DNA modifications in cardiac aging and disease is an understudied but important future research direction.

### Altered balance of activating and repressive histone modifications

Histone post-translational modifications (PTMs) represent another epigenetic mechanism to control gene expression. A core octamer comprising two copies each of H2A, H2B, H3, and H4 histones wraps 147 bp of DNA to form the basic unit of chromatin, the nucleosome. Linker histone H1 binds to linker DNA at the entry and exit sites of DNA on nucleosomes to form the next level of recurring chromatin structural unit, the chromatosome^[114]^. Core and linker histones are modified by diverse PTMs such as acetylation, methylation, ubiquitylation, phosphorylation, *etc*. primarily on the unstructured tail regions (although there are many core modifications) and regulate activation or repression of gene expression via opening and closing of chromatin structure in a heritable fashion^[115]^. Table 1 provides an overview of known functions of specific histone modifications from the literature. Active or open chromatin is referred to as euchromatin, and inactive, closed chromatin is called heterochromatin. An existing notion in senescence studies points towards the progressive euchromatinization of the genome with concomitant loss of repressive modifications.

A genome-wide investigation of 7 histone PTMs, lysine 9 acetylation on histone H3 (H3K9ac), H3K27ac, H3K79me2, H3K4me3, H3K9me2, H3K9me3, and H3K27me3 (“me” indicating methylation) in cardiomyocytes isolated from normal and pressure-overloaded hearts revealed a subset of hypertrophy-associated genes that follow the conventional histone code, i.e., a mutually exclusive enrichment of activating (H3K9ac, H3K27ac, H3K79me2, and H3K4me3) and repressive (H3K9me2, H3K9me3, and H3K27me3) modifications. Additionally, this study identified a network of ~9000 putative active enhancers in the hypertrophic heart that might correlate to disease pathology, suggesting that histone PTMs regulate the gene network involved in this process^[116]^.

A cross-tissue analysis of chromatin marks (H3K4me3 and H3K27ac) revealed a clear separation in the RNA and chromatin profiles of young, middle-aged, and old hearts. Importantly, these age-related chromatin features included H3K4me3 and H3K27ac intensity and H3K4me3 breadth, which was previously shown to be linked to transcriptional consistency and high expression output required for maintenance of cell identity. Both dynamic features (such as enhancer score and H3K4me3 breadth) and static features (such as H3K4me3 promoter intensity and H3K4me3 domain breadth in young tissue) were key predictors of age^[117]^.

Studies focusing on the repressive H3K9 methylation, specifically H3K9me2, revealed that it promotes the reexpression of fetal genes during pathological cardiac hypertrophy. Downregulation of the H3K9 dimethyltransferases EHMT1/2 by miR-217 leads to loss of H3K9me2 over the promoters of fetal heart genes such as atrial natriuretic peptide (*Nppa*), brain natriuretic peptide (*Nppb*), and *Myh7* in cardiomyocytes^[118]^. Knockout or overexpression of the H3K9 trimethyl demethylase JMJD2A (or KDM4A) had no overt cardiac phenotype but exhibited an altered response to stress. For example, overexpression of JMJD2A resulted in exacerbated cardiac hypertrophy while its inactivation was protective after aortic constriction^[119]^. These results suggest that histone H3K9 repressive modifications play a critical role in suppressing a cardiac stress response that may also be occurring during aging, although it remains to be explicitly tested.

Histone acetylation is a very dynamic histone PTM and is regulated by histone acetyltransferases and histone deacetylases (HDACs). Loss of HDAC1, 2, 3, 5, and 9 results in exacerbated cardiac hypertrophy and, in some cases, neonatal lethality or a shortened lifespan^[120–122]^. Sirtuins (SIRT1–7) are a family of nicotinamide adenine dinucleotide (NAD+)-dependent class III HDACs that have established protective roles in lifespan regulation in multiple species. However, both SIRT1 and NAD+ levels decline during aging^[123]^. Furthermore, loss of SIRT1 interferes with angiogenesis and neovascularization after ischemia due to aberrant acetylation of FOXO1, potentiating its anti-angiogenic function^[124]^. Conversely, overexpression of SIRT1 has many beneficial effects on endothelial cell function, including increased migration^[124]^, decreased endothelial progenitor cell senescence^[125]^, and reduced vascular oxidative stress and inflammation via inhibition of NFκB and PARP^[126]^. While SIRT1 also acts on histones, this aspect of regulation remains unexplored.

Available studies taken together confirm that histone modifications contribute to CVD, with the direction of changes similar to that observed during senescence. There is a pronounced shift in the balance characterized by reduced repressive marks, especially over repeat elements and increased active modifications. However, due to the paucity of direct work in aged tissue and the lack of integrative analysis, the exact mechanisms remain to be elucidated.

### A core TF network in aging

A multi-omic (DNA methylome, transcriptome, and epigenome) profiling and integrative analyses of the aging murine heart, liver, and quadriceps muscle identified some common and unique aging footprints across tissues. As a note, this study primarily performed a gene-centric analysis, and therefore changes over features such as enhancers or repeat regions were not analyzed. In the heart, the DNA methylome was the primary epigenetic signature that changed around transcription start sites (TSSs) with approximately an equal number of hypo- and hyper-methylated CpGs. Although more subtle, H3K27ac enrichment increased in the ~5 Kb region around the TSSs, while the H3K27me3 signal decreased. A TF motif enrichment analysis around promoters of genes up- or down-regulated in the heart during aging revealed that TF motifs enriched in genes that increase expression are also enriched in genes that have an increase in H3K27ac and a decrease in H3K27me3. Interestingly, a few TFs common to all three tissues were enriched in upregulated genes and genes with increases in H3K27ac and decreases in H3K27me3 in the heart. These TFs belong to the zinc finger of the cerebellum (Zic) family of factors. Conversely, HMGA1 binds to genes that are downregulated with age. Importantly, the expression of these TFs in humans is altered during aging, and epidemiological studies suggest a link between the altered expression of some of these TFs and the mother’s age^[127]^. These results suggest that a common set of epigenetic “master” regulators may be responsible for driving some of the key transcriptomic changes in aging.

### Increased transcriptional noise in the aging heart

Single-cell studies have emphasized the presence of heterogeneity and variability within tumors, complex tissues, and surprisingly even overtly pure cell populations. For example, an early study with purified cardiomyocytes isolated from fresh young and old mice hearts revealed increased gene expression variability in old cells^[128]^. The authors of the study attributed this increased variability to the stochastic nature of the aging process contributed by DNA damage and accumulating somatic mutations. Indeed, mouse embryonic fibroblasts treated with hydrogen peroxide showed a similar increase in expression variability. A more recent comprehensive single-cell atlas (Tabula Muris Senis^[129]^) of multiple mouse tissues, including heart and aorta, is available but begs for a deeper dive into the dataset to enable the discovery of age-related changes specific to the cardiovascular system.

### Non-coding RNA in cardiovascular aging

The vast majority of the genome is not translated into proteins but rather serves either as cis-regulatory elements or mediates post-transcriptional gene regulation^[130]^. These non-coding areas of the genome encode small non-coding RNAs (< 200 nucleotides) or long non-coding RNAs (> 200 nucleotides). Small non-coding RNAs mainly comprise micro-RNAs (miRNAs), piwi-interacting RNAs (piRNAs), transfer RNAs (tRNAs), small nuclear RNAs (snRNAs), small nucleolar RNA (snoRNAs), *etc*. Long non-coding RNAs can be either linear (lncRNAs) or circular (circRNAs). Non-coding RNAs have long been implicated in senescence and aging, with several studies conducted in the context of cardiovascular aging^[131,132]^.

miRNAs and circRNAs present reliable biomarkers of aging due to their stability in circulation and conservation across species. miR-21 is a particularly well-characterized miRNA targeting SPRY1, a potent inhibitor of the ERK-MAPK pathway. miR-21 increases in cardiofibroblasts of the failing heart, augmenting ERK-MAP kinase activity impacting interstitial fibrosis and cardiac hypertrophy^[133]^. In a study profiling miRNAs in the heart of neonatal, 1 month, 6 months and 19 months old mice, miR-22, which targets osteoglycin, was found to be robustly upregulated. miR-22 overexpression induced senescence and promoted the migration of cardiac fibroblasts^[134]^. miR-34a is induced in aging cardiomyocytes where it targets PNUTS, a cardioprotective protein that otherwise reduces age-associated cardiomyocyte cell death^[135]^. Interestingly, miR-34a, through the targeting of a different protein, SIRT1 (discussed above), induces endothelial and VSMC senescence and proinflammatory SASP expression^[136,137]^. SIRT1 is also targeted by miR-217 in ECs, where it induces premature senescence and leads to an impairment in angiogenesis via modulation of FOXO1 and nitric oxide synthase acetylation^[138]^. Transcriptomic analysis of aortic tissue in old mice revealed miR-29 upregulation and the concomitant downregulation of many ECM components that in turn sensitizes the aorta to aneurysm formation^[139]^. In contrast, a number of other miRNAs (miR-18, miR-19, miR-17–3p, miR-92, reviewed in^[140]^) are reduced in expression during aging, specifically elevating their targets to affect cardiovascular aging and disease.

CircRNAs impact transcription by acting as sponges of miRNA and RNA binding proteins (RBPs) or serving as scaffolds for assembly of larger complexes^[141]^. Many circRNAs are altered in expression upon hypoxic injury or myocardial infarction (reviewed extensively in^[142]^); a few relevant to aging are discussed here. circFoxo3 is generated from the *Foxo3* transcript and was shown to be overexpressed in the aged hearts of mice and humans and correlated to senescence markers. CircFoxo3 is localized to the cytoplasm where it retains several anti-senescence proteins such as ID1, E2F1, FAK, and HIF1α, thus siphoning their activity away from the nucleus^[143]^. In addition, a circRNA produced from the senescence/aging relevant *Cdkn2b* locus called Antisense non-coding RNA in the INK4 locus (circANRIL) correlates with the expression of its linear RNA and confers atheroprotection. circANRIL binds to PES1, a 60S-preribosomal assembly factor, impairs ribosome biogenesis and thereby induces nucleolar stress and apoptosis in atherogenic VSMCs and macrophages^[144,145]^. Unlike miRNAs that are well studied in aged hearts and vasculature, key age-related circRNAs remain to be profiled in detail.

lncRNAs, unlike miRNAs, are not well conserved across species, and therefore their targets and functions should be interpreted with caution. Nevertheless, numerous studies have evaluated the role of lncRNAs in cardiovascular aging and disease (reviewed in^[146]^). lncRNAs are highly versatile, serving as expression signals to trigger a response, competitive endogenous RNAs, guides to direct factors to specific genomic locations, scaffolds for RBPs, or mediators of chromatin looping^[146]^. For example, *Mhrt*, an antisense lncRNA produced from the region between *Myh6* and *Myh7*, interferes with the switch to fetal *Myh7* expression in hypertrophic hearts. *Mhrt* antagonizes BRG1 function by interacting with its helicase domain and inhibiting chromatin targeting (see next section)^[147]^. *Chaer*, another lncRNA mediating cardiac hypertrophy, interacts with PRC2 subunits and thereby inhibits the repression of cardiac hypertrophy-related genes^[148]^. *Meg3* expression is upregulated in senescent HUVEC (endothelial) cells and in the aging cardiovascular system, where it also targets PRC2 components to assert a pro-aging function in aging vasculature^[149]^. An RNA-seq study in porcine cardiac muscle revealed 4 lncRNAs that were consistently expressed during aging. Ontology analysis of the target genes of these lncRNAs was significantly enriched for negative regulation of myotube differentiation and muscle contraction, suggesting that the lncRNAs likely interfere with the normal muscle physiology^[150]^.

There are numerous other examples of non-coding RNA functions in cardiovascular aging that are beyond the scope of this review. However, it is interesting to note that many of them target epigenetic enzymes or TFs and therefore may directly and pervasively impact the epigenome.

### ATP-dependent chromatin remodeling in the diseased heart

ATP-dependent chromatin remodeling complexes are large multi-subunit molecular machines that utilize ATP to reposition or evict nucleosomes or exchange histones to alter chromatin structure. The BRG1/BRM-associated factor (BAF) chromatin remodeling complexes are comprised of either brahma or brahma-related gene 1 (BRG1) catalytic subunits along with several other accessory proteins. BAF complexes are critical for heart development and disease pathogenesis. For example, BRG1 plays opposing roles at the *Myh6* and *Myh7* loci: in embryos, it interacts with HDACs and poly (ADP ribose) polymerase 1 (PARP1) to repress the adult-specific *Myh6* while activating fetal *Myh7*^[151]^*. Brg1* expression is lost in cardiomyocytes but reactivated in hypertrophic hearts. It sequentially recruits G9a and then DNMT3 to deposit H3K9me2 and 5mC at the *Myh6* promoter impairing cardiac contraction^[152]^. Thus, a complex interplay of repressors and co-repressors recruited by BRG1 in injured hearts activates fetal *Myh7* while interfering with adult *Myh6* expression. Cardiac regeneration (as may be promoted by injury) requires BRG1 not only to suppress *Myh6* but also to increase the expression of pro-proliferative *Bmp10* and *Cdkn1c* genes. Although not directly tested in aging hearts, reactivation of *Brg1* is a plausible mechanism to promote repair. Finally, mutations in genes encoding BAF complex subunits have been associated with various cancers and congenital heart diseases^[153,154]^.

### Laminopathy and loss of heterochromatin lead to premature aging

Hutchinson Gilford Progeria Syndrome (HGPS) is a premature aging disorder attributed to a mutation in the lamin A (*LMNA*) gene that results in the production and incorporation of a truncated version of lamin A called progerin in the nuclear membrane. This misincorporation grossly disrupts the nuclear lamina and lamina-associated heterochromatin and pro-senescence/pro-aging gene expression changes. Surprisingly, HGPS patients usually die in their teens from atherosclerosis and CVD complications suggesting strong links between chromatin dysregulation and CVD events in these patients. Interestingly, vascular progerin production and its progressive increase with age have also been noted in normal individuals, and there are many common histological features in the vasculature of HGPS and geriatric subjects^[155]^. Additionally, fibroblasts isolated from HGPS patients undergo premature senescence, and in iPSC models of HGPS, many epigenetic changes noted mimic those found in *in vitro* models of cellular senescence. For example, there is a reduction in repressive histone modifications, H3K27me3 and H3K9me3, loss of EZH2, and derepression of LINE elements^[156,157]^. This evidence suggests that epigenetic alterations, particularly those found in senescent cells, may drive some of the key CVD pathologies in HGPS. Indeed, selective clearance of naturally occurring p16 positive senescent cells in the heart and senescent foam macrophages at atherosclerotic lesions by senolytics can ameliorate disease symptoms^[33,158]^.

**Figure 2.**
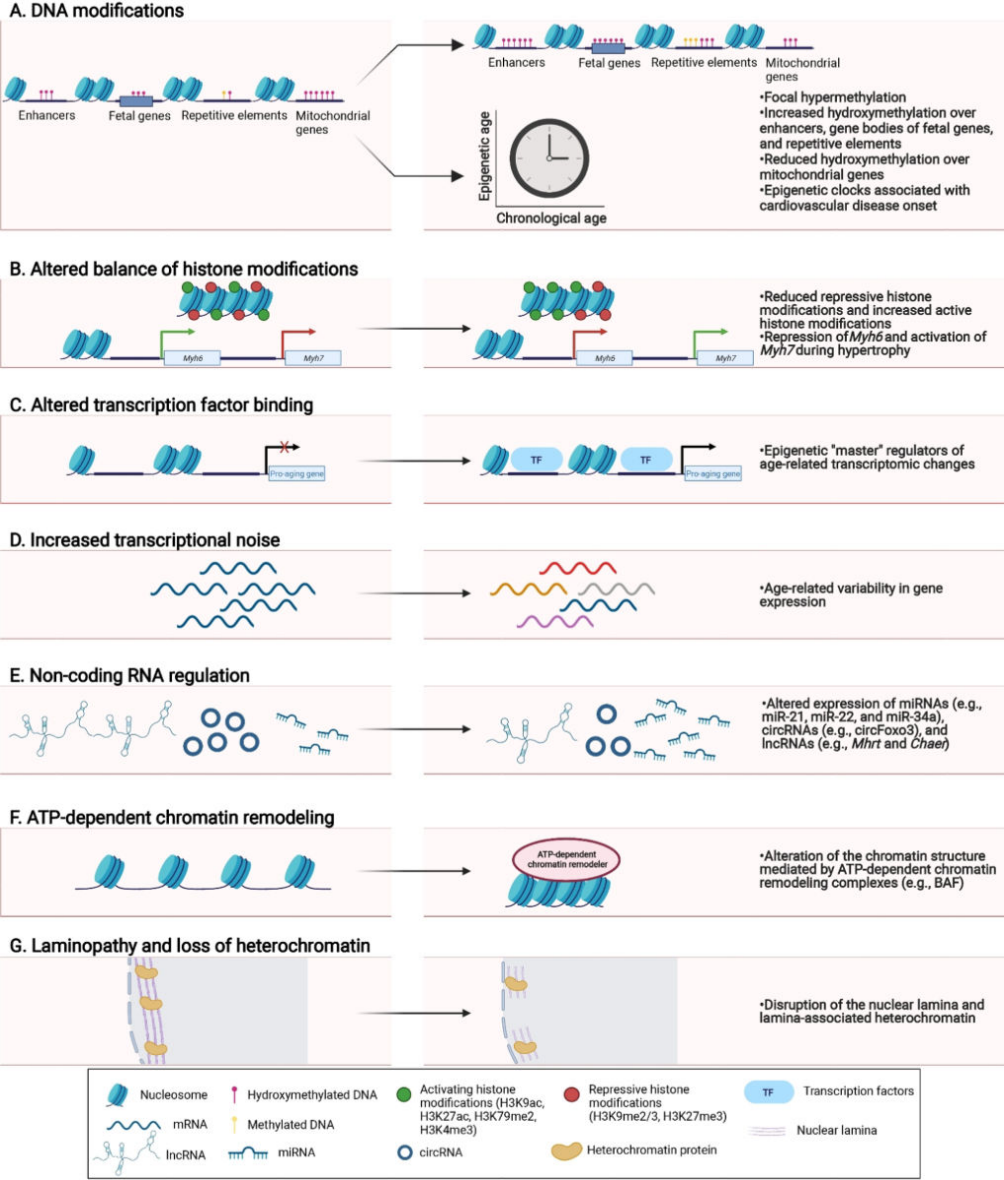
Epigenetic mechanisms involved in cardiovascular aging and disease. Several epigenetic changes are documented in cardiovascular aging and disease, including (A) DNA modifications (5-methylcytosine is also used in epigenetic clocks and associated with cardiovascular disease onset); (B) altered balance of active and repressive histone marks; (C) alterations to transcription factor binding; (D) transcriptional changes; (E) altered expression of non-coding RNAs; (F) chromatin remodeling; and (G) laminopathy and loss of heterochromatin.

Figure 2 summarizes the key concepts of epigenetic regulation impacting CVD: DNA modifications [Figure 2A], histone modifications [Figure 2B], TF binding [Figure 2C], altered gene expression [Figure 2D], non-coding RNAs [Figure 2E], chromatin remodeling [Figure 2F] and lamina disorganization [Figure 2G] as derived from models of heart failure, and limitedly, aged tissues.

## FUTURE PERSPECTIVES AND PROVOCATIVE THERAPIES FOR AGE-RELATED CVD

With the advent of better health monitoring, chemotherapies, vaccines, and rehabilitation programs, human life expectancy has increased and will continue to increase in the next few decades. This means that the number of people > 65 years of age will comprise 20% or more of the population by the next decade. Unfortunately, CVD will remain one of the top causes of death among older individuals, surpassing neurodegenerative diseases and cancers, suggesting that the cardiovascular system is especially prone to the chronic deleterious changes that come with age. Until recently, age was thought to be a largely unmodifiable feature of life, but the innovations of the longevity biotechnology field are on a trajectory to change this outcome. Thus, now is the time to identify key mechanisms contributing to heart disease in the elderly to design and rapid testing of breakthrough therapeutics.

Several notable interventions which directly or indirectly remodel the epigenome hold promise in ameliorating CVD. Preclinical studies in mouse models have already shown the efficacy of senolytics in countering the deleterious effects of cardiac dysfunction, vascular dysfunction, and calcification^[33,34,159,160]^. Senostatics that reduce the SASP without eliminating senescent cells, which carry the risk of fibrosis, might also show benefits but have not been directly tested. Potential SASP modulators include glucocorticoids^[161]^, rapamycin^[162]^, metformin^[163]^ and CR/CR mimetics^[164]^. Although the exact mechanisms underlying age reversal are lacking, these molecules have a profound effect on the epigenome (reviewed in^[87,88,165]^). Some direct effects of epigenome remodeling are exemplified by enzymes such as MLL1^[166]^ and BRD4^[167]^, which are critical regulators of *SASP* genes. Additional synthetic therapeutics that could potentially modulate senescence or SASP include locked nucleic acids, anti-miRs, and other antisense oligonucleotides that block non-coding RNA activity and/or target them for degradation^[168]^. Overall, given the predominance of senescent cell function in CVD, targeting them is a viable option to treat age-related cardiac dysfunction.

Another targetable cell type in CVD is the quiescent cardiomyocyte and fibroblast populations that comprise most adult heart tissue. Unlike senescent cells, quiescent cells are responsive to growth factors and apoptotic signals, making them more pliable for modulation. While neonatal cardiomyocytes are capable of proliferation and regeneration, this function declines rapidly in adults^[169]^. The cardiac stem cell theory was recently annulled following extensive fate-mapping data that clearly showed that non-myocytes could not produce new cardiomyocytes in the adult during homeostasis or following infarction^[170]^. Thus, an endogenous stem cell-centric therapy in CVD is contentious. However, the exogenous supply of cardiac progenitors produced from induced pluripotent stem cells could be explored but need careful testing. Another avenue to improve regeneration of adult cardiomyocytes is by inducing controlled proliferation, for example, by cyclical expression of Yamanaka factors. In a premature aging model carrying a *Lmna* mutation, cyclic induction of these pluripotency factors partially rescued the degeneration of VSMCs in the aortic arch compared to untreated mice, as indicated by an increase in the nuclei number. At the functional level, electrocardiographic analysis showed that there was also a partial rescue of bradycardia in the treated mice compared to controls. Yamanaka factors induce reprogramming by changing the histone modification landscape, specifically restoring H3K9me3 and H4K20me3 to youthful levels^[171]^.

Pathological, activated cardiac fibroblasts (as opposed to quiescent fibroblasts) are induced following cardiac injury and can cause excessive fibrosis. These activated fibroblasts have been shown to have a unique gene expression signature, prominently the upregulation of fibroblast activation protein, which was targeted to eliminate them by chimeric antigen receptor (CAR)-T cell therapy^[172]^ selectively. We propose that a similar survey of the transcriptome and epigenome can discover neoantigens on aged tissues, which can then be exploited for immunotherapy in CVD.

Of note, most of the studies discussed in this review use mouse models of heart failure or cardiac disease to interrogate epigenetic features. However, the experimental rodent is usually an adult (2–4 months old) with a very different epigenomic landscape than older animals, who present the most risk for disease. These models thus may accurately capture acute pathological changes while completely missing the contribution to disease of any long-term chronic effects such as systemic inflammation or global epigenetic changes. Conversely, studies that focused on interventions that extend lifespan rarely measured whether the cardiovascular function was improved^[173]^. Collectively, the field must embrace naturally aged mice models and impose the inclusion of age as a biological variable to gain deeper insight into the etiology of age-related cardiac dysfunction and disease.

## CONCLUDING REMARKS

In the studies that have considered both the perspective of aging and cardiovascular health, we have accumulated important insights into the epigenetic mechanisms of cardiovascular aging that we describe in this review. Genomic regions that are targeted during aging include repeat elements and lamina-bound heterochromatin, developmental gene promoters, polycomb targets, and stress response genes. These regions also show prominent changes in DNA modifications. The histone code itself is unaltered, but specific master TFs co-opt epigenetic enzymes and chromatin architectural proteins to promote a disease phenotype. Concordant with changes in DNA and histone modifications, the coding, and non-coding transcriptome is also significantly altered, impacting cardiac function and vascular physiology. Further studies will illuminate more precise roles of epigenetic factors that can ultimately be exploited to design novel therapeutics.

## Figures and Tables

**Table 1.** Histone modifications involved in cardiovascular aging and disease

	Function	Enzymes(s)	Involvement in cardiovascular aging/disease	Model	Ref.
**H3K9ac**	Active promoter	KAT2A/2B, KDM4A-D, KDM3A-B	Positively associated with transcription in hypertrophic cardiomyocytes	*Homo sapiens, Mus musculus*	[116,174]
**H3K27ac**	Active promoter and enhancer	KAT3A-B, various HDACs	Increased at promoters of age-related genes	*Mus musculus*	[127,175]
**H3K79me2**	Body of active genes	KMT4	Associated with cardiac hypertrophy	*Mus musculus*	[116,176]
**H3K4me3**	Active promoters	KMT2F, KMT2G, KMT2A, KMT2D, KMT8B, KDM5A-D	Domain breadth increases in the aging heart	*Homo sapiens, Mus musculus, Drosophila melanogaster, Caenorhabditis elegans, Arabidopsis thaliana,* and *Saccharomyces cerevisiae*	[115,117]
**H3K9me2**	Nuclear lamina-associated heterochromatin	KMT1C, KMT1D, KDM3A-B, KDM4A-E, KDM7B	Reduced in pathological cardiac hypertrophy	*Mus musculus*	[118,177]
**H3K9me3**	Constitutive heterochromatin, repeat elements	KMT1A-B, KDM4A-E	JMJD2A-mediated demethylation is associated with cardiac hypertrophy and heart failure. Reduced in HGPS patients	*Homo sapiens, Mus musculus*	[119,156,171,178]
**H3K27me3**	Facultative heterochromatin	KMT6A-B, KDM6A-B	Reduced in HPGS patients	*Homo sapiens*	[157,179]
**H4K20me3**	Heterochromatin	KMT5B-C, KDM7C	Increased in aging mice fibroblasts	*Homo sapiens*	[171,180]
